# Management of atypical femoral fracture: a scoping review and comprehensive algorithm

**DOI:** 10.1186/s12891-016-1086-8

**Published:** 2016-05-23

**Authors:** Giuseppe Toro, Cristina Ojeda-Thies, Giampiero Calabrò, Gabriella Toro, Antimo Moretti, Guillermo Martínez-Díaz Guerra, Pedro Caba-Doussoux, Giovanni Iolascon

**Affiliations:** Department of Medical and Surgical Specialties and Dentistry, Second University of Naples, Via De Crecchio, 4, 80138 Naples, Italy; Trauma Unit, Department of Orthopaedic Surgery and Traumatology, Hospital Universitario 12 de Octubre, Madrid, Spain; Unit of Orthopaedics and Traumatology, Villa Malta Hospital, Sarno, Italy; Unit of Radiology, Santa Maria della Speranza Hospital, Battipaglia, Italy; Metabolic Bone Disease Unit, Department of Endocrinology, Hospital Universitario 12 de Octubre, Madrid, Spain

## Abstract

**Background:**

Atypical femoral fractures (AFF) are a rare type of femoral stress fracture recently described, potentially associated with prolonged bisphosphonate therapy. Evidence-based recommendations regarding diagnosis and management of these fractures are scarce. The purpose of this study is to propose an algorithm for the diagnosis and management of AFF.

**Methods:**

We performed a PubMed search of the last ten years using the keywords “atypical femoral fractures” and identified further articles through an evaluation of the publications cited in these articles. Relevant studies were included by agreement between researchers, depending on their specialization. Pertinent points of debate were discussed based on the available literature, allowing for consensus regarding the proposed management algorithm.

**Results:**

Using a systematic approach we performed a scoping review that included a total of 137 articles.

**Conclusions:**

A practical guide for diagnosis and management of AFF based on the current concepts is proposed. In spite of the impressive large volume of published literature available since AFF were initially identified, the level of evidence is mostly poor, in particular regarding treatment choice. Therefore, further studies are required.

## Background

The World Health Organization considers osteoporosis to be second only to cardiovascular diseases as a critical health problem, due to the high prevalence, costs and effect on quality of life caused by osteoporotic fractures [1, 2]. Osteoporotic fractures account for more disability and life-years lost (DALYs) than for all sites of cancer, with the exception of lung cancers [3]. Proximal femoral fractures are responsible for the most serious consequences of osteoporosis, due to their elevated incidence, as well as the hospitalization costs and disability following these fractures, with a financial burden equivalent to cardiovascular disease [4]. The number of proximal femoral fractures is expected to increase worldwide due to ageing of the population [5, 6].

Bisphosphonates (BPs) are the most commonly prescribed medication to reduce bone resorption and prevent osteoporotic fractures [7–10]. Bisphosphonate therapy has been associated with adverse events, such as osteonecrosis of the jaw and atypical femoral fractures (AFF) [11–13]. The latter are tensile stress fractures with defined radiographic features involving the femur from subtrochanteric to supracondylar flare. The American Society for Bone and Mineral Research (ASBMR) proposed a set of specific criteria in order to identify a case as an atypical femoral fracture (Table 1) [12]. These criteria should differentiate AFF from “typical” femoral subtrochanteric or diaphyseal fracture. However, a clear definition of which should be a “typical” femoral fracture is not given. Osteoporotic fractures are associated with low energy trauma and usually have a long oblique or spiral pattern. High-energy trauma fractures are characterized by a typical complex pattern, with an increased degree of displacement and comminution [14]. A short oblique or transverse fracture of the femoral shaft can be due to a direct high-energy impact, such as dashboard injuries; posterior or medial third wedge fragment is commonly associated with this pattern.

**Table 1:** 2013 ASBMR task force criteria of atypical femoral fractures

ASBMR criteria: Four of five major criteria should be observed; additional minor criteria are not necessary for diagnosis but could be observed in association to the major criteria.	
Major	- The fracture is associated with minimal or no trauma, as in a fall from a standing height or less - The fracture line originates at the lateral cortex and is substantially transverse in its orientation, although it may become oblique as it progresses medially across the femur - Complete fractures extend through both cortices and may be associated with a medial spike; incomplete fractures involve only the lateral cortex - The fracture is noncomminuted or minimally comminuted - Localized periosteal or endosteal thickening of the lateral cortex is present at the fracture site (“beaking” or “flaring”)
Minor criteria	- Generalized increase in cortical thickness of the femoral diaphyses - Unilateral or bilateral prodromal symptoms such as dull or aching pain in the groin or thigh - Bilateral incomplete or complete femoral diaphysis fractures - Delayed fracture healing

Fractures of the femoral neck, intertrochanteric fractures with spiral subtrochanteric extension, periprosthetic fractures, and pathological fractures associated with primary or metastatic bone tumors and miscellaneous bone diseases (eg, Paget’s disease, fibrous dysplasia) are excluded [12]

**Fig. 1:**
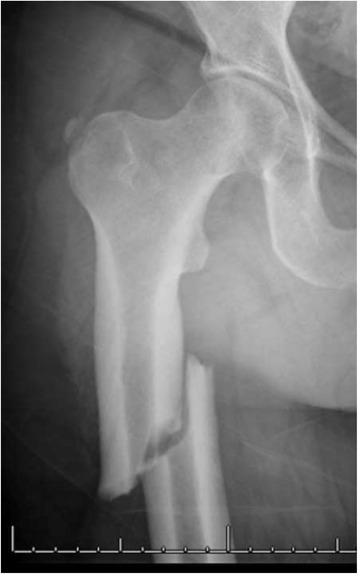
Radiograph of a patient with a complete AFF. Note the substantially transverse orientation of the fracture line at the lateral cortex, the medial spike and the generalized cortical thickening

**Fig. 2:**
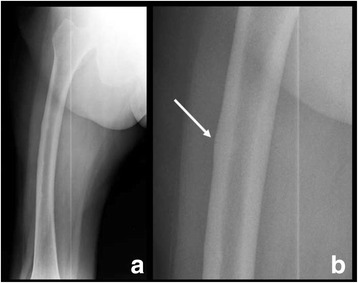
Radiograph of a patient with an incomplete AFF (**a** and **b**, detail). **a**. Note the femoral bowing. **b**. Note the location of the fracture line on the lateral cortex,and the focal cortical thickening at the fracture site

Barcsa et al. first coined the term “atypical” in 1978 in his description of fatigue fractures [15], but the first report of bisphosphonate-related femoral fractures was published by Odvina et al. in 2005, who suggested a prominent pathogenic role of severe bone turnover suppression caused by these drugs [16]. Since then, numerous case reports and case series, as well as registry-based studies, described atypical femoral fractures following low-energy trauma and associated with prolonged use of BPs [17–24]. Growing concern brought the ASBMR to assemble a specific task force in order to resolve the controversy over this issue and publish a position paper in 2010 [25]. A second report was published by the ASBMR task force in 2013 in order to review the major reports that had been published since 2010, focusing on three aspects of atypical femur fractures: their epidemiology, pathogenesis, and medical management [12]. Figures 1 and 2 show the characteristic patterns of both complete and incomplete AFF.

Another issue concerning the management of AFF is fracture healing, that seem to depend on several factors, including pattern of fracture line, particularly short oblique or transverse, varus malreduction at the fracture site, and suppression of bone turnover [26, 27]. Therefore, the treatment of AFF presents as a challenge for the orthopedic surgeon [13, 28–31].

The goal of this scoping review is to propose a practical diagnostic and treatment algorithm for AFF, in order to help orthopedic surgeons in the management of AFF.

## Methods

This scoping review began by creating a research group formed by five specialists in orthopedic surgery and traumatology, a physiatrist, an endocrinologist, and a radiologist. All members have expertise in the field of metabolic bone disease. The research team performing the scoping review discussed during a preliminary meeting which were the open issues about the appropriate management for atypical femoral fractures. After that a secret voting session was performed and it was decided that all the issues that got more than 50 % of votes would have been addressed. Therefore, the 4 major questions that we are going to clarify with this scoping review are:

1. How do we make diagnosis of AFF?
2. How to perform the evaluation of bone turnover in patients with AFF?
3. How to manage the contralateral femur in patients with AFF?
4. What is the decision making process when a AFF occurs?.

According to Arksey’s recommendations the scoping review process included the following six key steps: 1) identification of the research question, 2) identification of relevant studies, 3) study selection, 4) charting the data, 5) collecting, summarizing, and reporting the results and 6) consultation exercise [32].

To define atypical femoral fractures, we used the criteria proposed by the ASBMR task force in 2010 and in 2013 [12, 25]. The research protocol was based on a PubMed search between August 2004 and August 2015, using “atypical femoral fracture” as keyword. Two independent researchers performed a basic review of the titles and abstracts; in case of unrecoverable abstracts, the full text was directly reviewed. All articles in English, Spanish or Italian language were considered eligible for the review. Relevant articles were marked with consensus between researchers. References cited in the included articles were also reviewed. The identification of relevant articles to be included in the review were performed following these criteria:

1. articles published between August 2004 and August 2015. This period was chosen because, to our knowledge, the first report of the possible association between BPs use and femoral shaft fractures was published by Odvina et al. in the end of 2004 [16].
2. all the articles where the AFF was identified following criteria that matched those defined by the ASBMR, including articles published before the statements of ASBMR.
3. articles who did not met the ASBMR criteria but considered relevant by researchers in order to respond to one or more specific research topics (i.e. imaging of stress fractures).

The flux of information was organized and analyzed considering the 4 major topics previously mentioned. Finally, consensus was obtained on key points drawn from each study.

All authors declare that they have no competing interests to disclose.

## Results

**Fig. 3:**
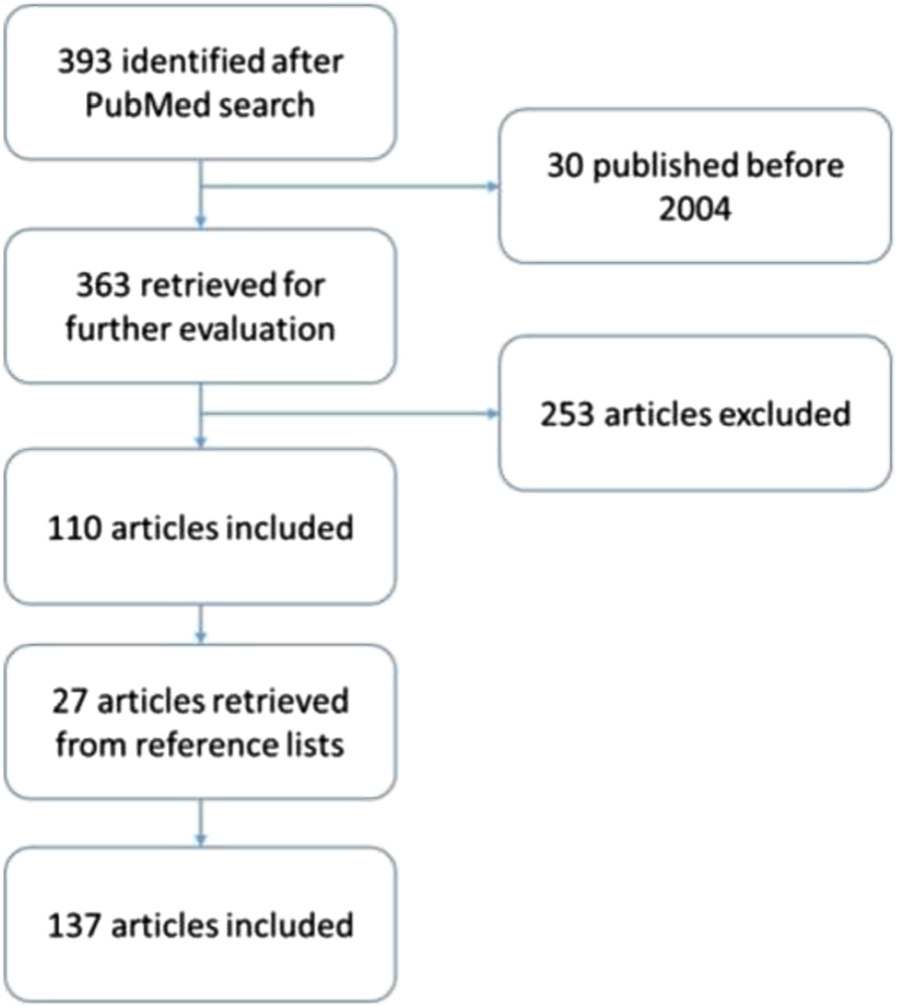
Summary of the article selection process

We identified 393 articles from our initial PubMed search for “atypical femoral fracture”. Of these, 363 were published since August 2004 and were therefore initially considered and discussed. A total of 137 articles met the inclusion criteria and were therefore included and discussed in this scoping review (Fig. 3). The studies included were classified according to the study design as follows: 11 observational studies (3 registry cohort studies and 8 case-control studies), 33 case series studies, 79 case reports, and 14 reviews. The registry cohort studies, 1 based on administrative database of Medicare, 1 on National Swedish Patient Register, and 1 on National Danish Hospital Discharge Register, investigated about the association between BPs use and AFF. The case-control studies aimed to evaluate the risk of AFF among BPs users (1 study), to characterize the patients with AFF (3 studies), to examine the AFF pathogenesis (2 studies), to define surgical outcomes of patients who sustained an AFF (1 study), and to assess the diagnostic utility of DXA examination in individuals with AFF (1 study).

### Diagnosis of AFF

#### Clinical presentation of atypical femoral fractures

An adequate patient history and physical examination is essential to make a diagnosis of AFF, particularly in cases of incomplete fractures. Prodromal thigh or groin pain is common [14]. The FDA and the European Medicines Agency (EMA) recommend attention to the appearance of thigh/groin pain among long-term bisphosphonate users [14, 33–35]. In these patients thigh/groin pain could be prodromic of a subsequent fracture, that could occur 1 week to 2 years later [35]. Severe pain appearing suddenly after a history of chronic thigh or groin pain is considered to be pathognomonic of a complete fracture. Varus deformity of the lower limb or femoral bowing are also to be assessed as risk factors for AFF [36–39] (Table 2).

**Table 2:** Risk factors for atypical femoral fractures

- Long time and/or high compliant BPs user - Proton pump inhibitor or glucocorticoid use - Genu varus - Varus/bowed femur - Contralateral recent AFF - Collagen disease	

It is mandatory to investigate the patient’s history regarding prior and current medications as well as the mechanism of injury (Table 3). The latter, indeed, is one of the major diagnostic criteria of AFF, which occurs without prior trauma or following low-energy trauma, defined as a fall from a standing height or less [12]. Giusti et al. found an association between BPs-related AFF (BRAFF) and concomitant use of proton pump inhibitors (PPI) and glucocorticoids, but the mechanisms contributing to facilitate the occurrence AFF is not well known [35].

**Table 3:** Patient history and clinical findings following an atypical femoral fracture

Comorbidities: - Collagen diseases - Rheumatoid arthritis - Pulmonary diseases (asthma, other chronic pulmonary disease)	Medications: - Bisphosphonate or other antiresorptive therapy - Proton pomp inhibitors - Glucocorticoid therapy
Incomplete fractures: - Persistent groin or thigh pain	Complete fractures: - History of groin or thigh pain (not always) - Acute pain, limb shortening and swelling, ecchymosis

Other factors seem to be involved in pathogenesis of AFF even among BPs users (Table 4), such as demographic factors, race and age. Marcano et al. observed that patients with AFF were usually younger and more often of Asian origin than patients treated with BPs without AFF or patients with osteoporotic proximal femoral fractures [40]. The higher occurrence among Asians may be due to their geometrical features, as recently suggested by Oh et al. [41, 42]. Some diseases seem to be more common among patients with AFF. Collagen diseases are the most common comorbidity observed in AFF in the series described by Saita et al. [43]. On the other hand, Giusti et al. observed that chronic pulmonary disease, asthma, rheumatoid arthritis and diabetes were the most common comorbidities reported in AFF patients with the total number of comorbidities higher in patients with a subtrochanteric fractures than in those with diaphyseal fractures [35].

**Table 4:** Pathogenesis of atypical femoral fractures

AFF pathogenesis	
Reduced bone turnover	BPs and other powered antiresorptive drugs (i.e. denosumab)
Lower limb geometry	Large femoro-tibial alignment (genu varum) Bowed Femur Varus neck-shaft angle
Others	age, race, drugs, comorbidities

#### Imaging of atypical femoral fractures

Several imaging modalities are offered to the diagnosis of atypical femoral fractures. Standard x-rays of the femur in anteroposterior and lateral views, are usually able to identify the fracture and describe its pattern. The ASBMR task force defined the precise radiological characteristics for AFF (Table 1).

The definition of “substantially transverse” is a cause of concern. Some authors interpreted it as an angle of less than 30 degrees from a line drawn perpendicularly to the lateral femoral cortex [23, 44]. However, focal cortical thickening and a transverse fracture on the lateral side are the elements with the highest accuracy for diagnosis of AFF [45].

**Fig. 4:**
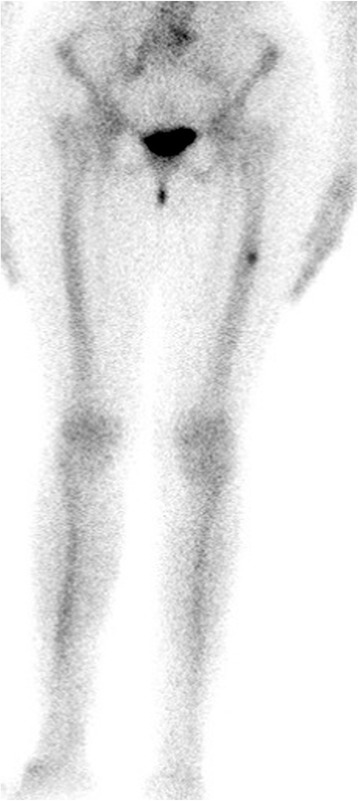
Bone scintigraphy of a case of incomplete AFF

**Fig. 5:**
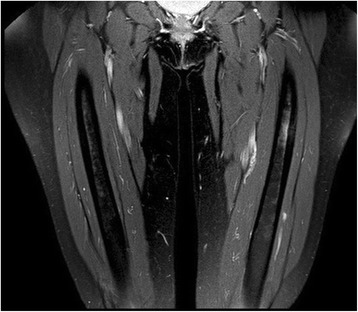
STIR-weighted MRI of the same case shown in Fig. 4. Note the increased fluid signal

Computed tomography (CT), magnetic resonance imaging (MRI) and other imaging modalities are of use, particularly in case of incomplete AFF [46]. CT is usually able to demonstrate abnormal bone texture and incomplete fractures [47]. MRI is the most sensitive and specific imaging modality to identify stress fractures, which present as an increased fluid signal [47]. Cortical thickening can also be observed in AFF [14]. Bone scintigraphy demonstrated a high ability to early individuate AFF [48]. Mild radiotracer uptake with endosteal thickening, along the lateral proximal diaphysis is considered a relatively specific finding in these fractures [49]. Figures 4 and 5 show the bone scintigraphy and MRI findings in an incomplete AFF.

Several authors have suggested that dual-energy x-ray absorptiometry (DXA) scans could be useful for the early detection of AFF [50–54]. The most common findings associated with AFF are focal cortical changes both periosteal and endosteal [53]. Therefore, McKenna et al. recommended extending DXA evaluation to the entire femur in chronic bisphosphonate users [52], considering the higher reliability to detect AFF in presence of prodromal signs [53].

### Evaluation of bone turnover

Theoretically, most of AFF should occur in low bone turnover, considering that the current pathogenic hypothesis for BPs-related AFF is an accumulation of microcracks in a “frozen” bone with a very low turnover caused by antiresorptive therapy [12, 25]. This hypothesis is corroborated by the observation of similar fracture patterns in congenital bone diseases characterized by low bone turnover, such as hypophosphatasia or pycnodysosthosis [55, 56].

Schilcher et al. studied the role of low bone turnover by performing a histological evaluation of eight cases of AFF, which showed signs of useless attempts of bone remodeling in order to heal the bone in the vicinity of the fracture gap [24]. Tjhia et al., using nanoindentation, showed higher resistance to plastic deformation and less heterogeneous elastic properties of bone tissue which could decrease resistance to propagation of microcracks in patients with severe suppressed bone turnover (SSBT), compared to osteoporotic and young individuals [57]. In a microindentation analysis, Güerri-Fernández et al. observed deteriorated mechanical proprieties of the bone in patients with AFF, whereas this was not observed among patient treated with BPs but without AFF; the authors suggested that BPs were not the only factors playing a role in the development of an AFF [58].

However, the ASBMR Task Force considers the evidence of the association between BPs use and AFF quite robust [12], with the fracture risk linked to longer duration and better adherence to therapy [59]. On the other hand, it must be underlined that the incidence of these fractures in BP users is extremely low and, not all AFF occur in BP users [60, 61].

### Management of the contralateral femur

AFF affect the contralateral leg in 28 % of cases [12], with the time between fractures ranging from 1 month to 4 years [35], but they can also be simultaneous [62]. Therefore, adequate study of the contralateral femur is mandatory, as recommended also by the EMA and the FDA [33, 34]. The evaluation of the contralateral femur should be done during the initial hospital stay, in order to quickly determine how to treat or to prevent the contralateral fracture. An X-ray exam of the entire contralateral femur is advisable, even if prodromal pain is absent [33].

### Decision making about treatment of the fractured femur

#### Conservative management

Conservative treatment is an option only in case of patient with incomplete fractures or severe comorbidities. It is mandatory to stop the ongoing antiresorptive therapy.

Patients who discontinued BPs therapy had a contralateral AFF incidence of 19.3 % in the following three years, compared with 41.2 % if the BPs were continued [14]. Schilcher et al. observed that the risk of AFF fell by 70 %/year after discontinuation of BPs [63]. Pharmacological treatment is essentially based on administration of calcium and vitamin D supplements, together with bone anabolic drugs such as teriparatide. This drug showed to be effective in promoting callus formation even in cases of nonunion [64]. Many case reports and case series have shown the effectiveness of teriparatide in both complete and incomplete AFF [65–74]. In a retrospective case-control study on the effect of teriparatide in 45 cases of AFF, Miyakoshi et al. observed a reduction of healing time and increased union rate [74].

Nonetheless, the results of conservative treatment are usually poor. Ha et al. published the results of 14 cases of AFF treated by observation and analgesics. Ten patients eventually needed a surgical treatment, and none of the 4 others had total pain relief or signs of complete healing [75]. Banffy et al. reported only one successful outcome in 12 conservatively treated incomplete AFF using a protocol consisting of partial weight bearing and observation [76]. However, other authors reported good results using conservative treatment protocols that included avoiding weight bearing, vitamin D and calcium supplementation, and bone forming agents, such as teriparatide or strontium ranelate [65, 66, 71, 74, 77–81]. The ASBMR task force recommendations define conservative treatment as limiting or avoiding weight bearing in addition to medical management of the underlying disorder [12, 25].

The ASBMR task force summarizes the medical strategy of AFF as follows: it is reasonable to discontinue BPs, adequate calcium and vitamin D intake should be ensured, and teriparatide should be considered for those who appear not to heal with conservative therapy [12].

#### Surgical management

Femoral subtrochanteric and shaft fractures are usually treated with intramedullary (IM) nailing or plating. IM nailing is the treatment of choice for most authors in both complete and incomplete AFF; in the latter, IM nailing is invoked as a preventive approach. The preference towards IM nailing is explained by the fact that endochondral repair is usually not achievable with a plate. When IM nailing is chosen, overreaming of the medullary canal by at least 2.5 mm larger than the nail diameter is recommended [25]. Several authors recommend the use of long cephalomedullary interlocking nails, considering that stress fractures usually occurred both above and involving an IM interlocking nail used to treat a prior AFF fracture [41, 82–85]. Extreme caution is advisable when performing IM nailing in very bowed or narrow femora, because of increased risk for distal fractures and diaphyseal comminution [29, 84]. Careful identification of the correct entry point is mandatory, as well as choosing a thinner nail [82].

Plate fixation could be a substitute in order to avoid the complications and technical difficulties associated with IM nailing [24, 69]. A long locking plate could be a good option when choosing plate fixation, particularly in the case of fractures associated with SSBT, in which healing by second intention through a more elastic construct may adequately stimulate fracture healing. Plate-and-screw constructs are however associated with a high complication rate in AFF [25, 29]. As a consequence, their use in AFF is more restricted than IM nailing, even if some reports have proven their reliability in selected incomplete and complete AFF [24, 69, 86, 87].

Two different case series studies showed that surgical outcomes were generally poorer than in patients with similar fractures not treated with antiresorptive drugs and were burdened by more complications, such as intraoperative fractures, hardware failure, non-union and delayed union [28, 29]. Of the 42 BP-associated femoral shaft fractures reported by Prasarn et al. the two most common complications were hardware failure (13 %) and intraoperative fracture (21 %) [29].

Another cause of concern is the effect of bisphosphonates on fracture healing. A recent systematic review observed that BPs use is associated with delayed union in fractures of the distal radius and humerus, even if the latter finding was reported only in one of the 16 articles included in the review. Moreover, due to the small number of patients included, the authors were unable to come to any conclusion regarding the role of BPs use on femoral fracture healing [88]. However, a database analysis of the FDA Adverse Event Reporting System (FAERS) concluded that healing problems of femoral fractures among BPs users were an unusual complication, considering that most cases were observed in AFF [30]. Egol et al. found that complete healing of BRAFF treated with intramedullary nails was delayed but generally reliable [26].The mean time to union for AFF ranges from 6 to 12 months [13, 26, 29, 35, 89], but cases of nonunion have been reported [68, 90].

## Discussion

**Fig. 6:**
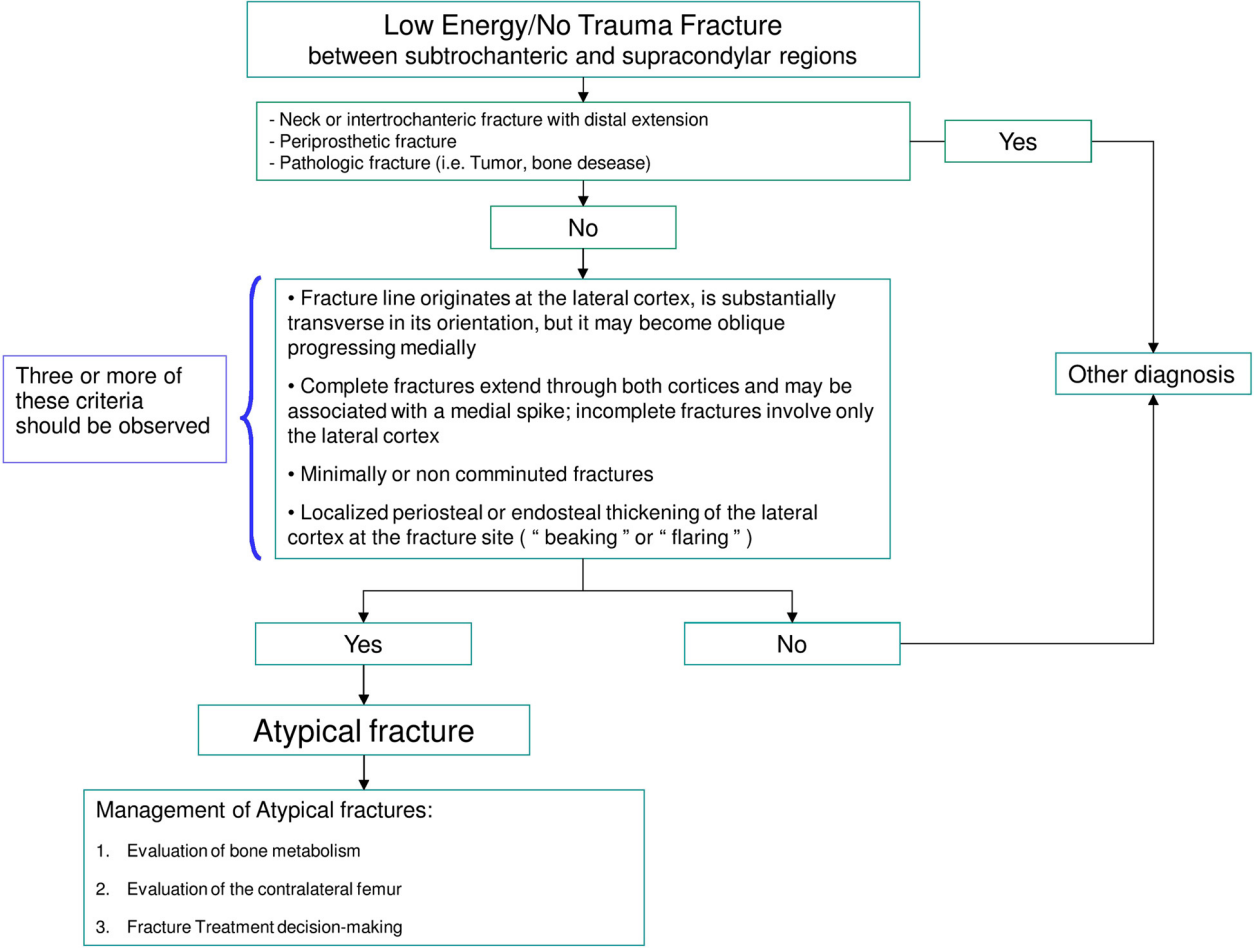
Diagnostic algorithm for atypical femoral fractures

The diagnosis of an AFF (Fig. 6) could be straightforward, if the ASBMR task force criteria are used for reference. If an AFF pattern is observed, fractures of the femoral neck or trochanteric area with distal extension, periprosthetic and pathologic fractures (both tumoral and miscellaneous bone disease such as Paget disease) should be excluded.

**Fig. 7:**
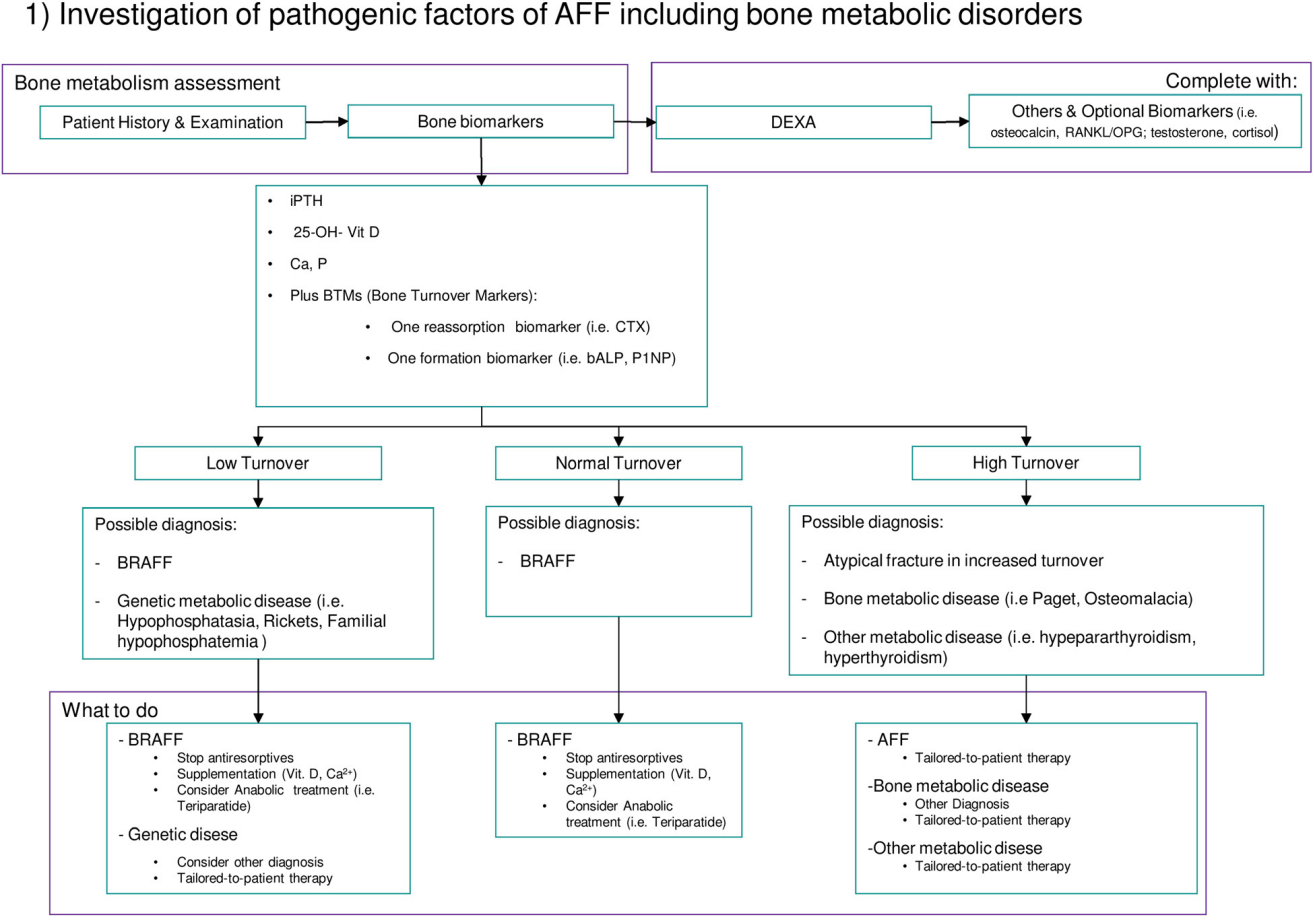
Evaluation of the state of bone metabolism and its correction. BRAFF = Bisphosphonate-related Atypical Femoral Fracture

**Fig. 8:**
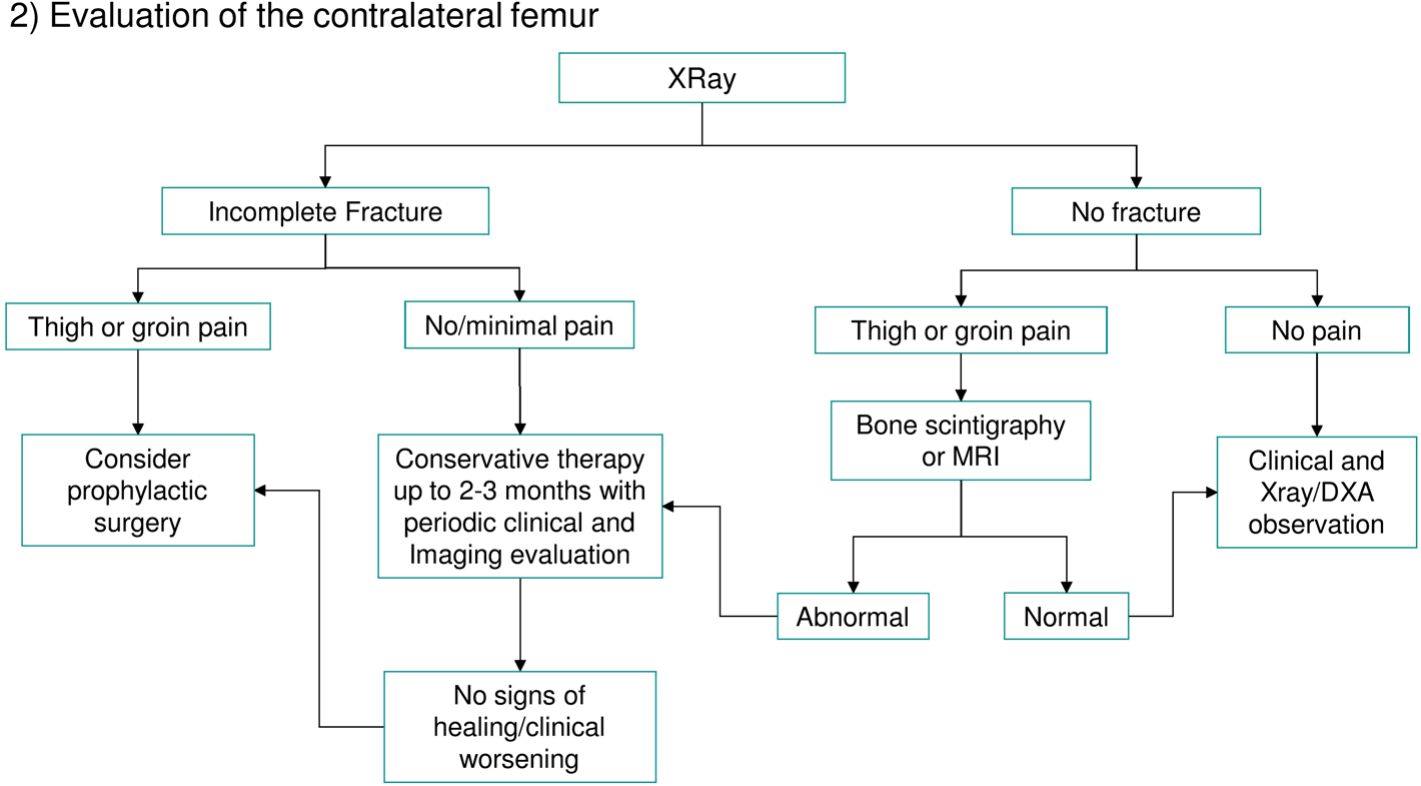
Evaluation of the contralateral femur

**Fig. 9:**
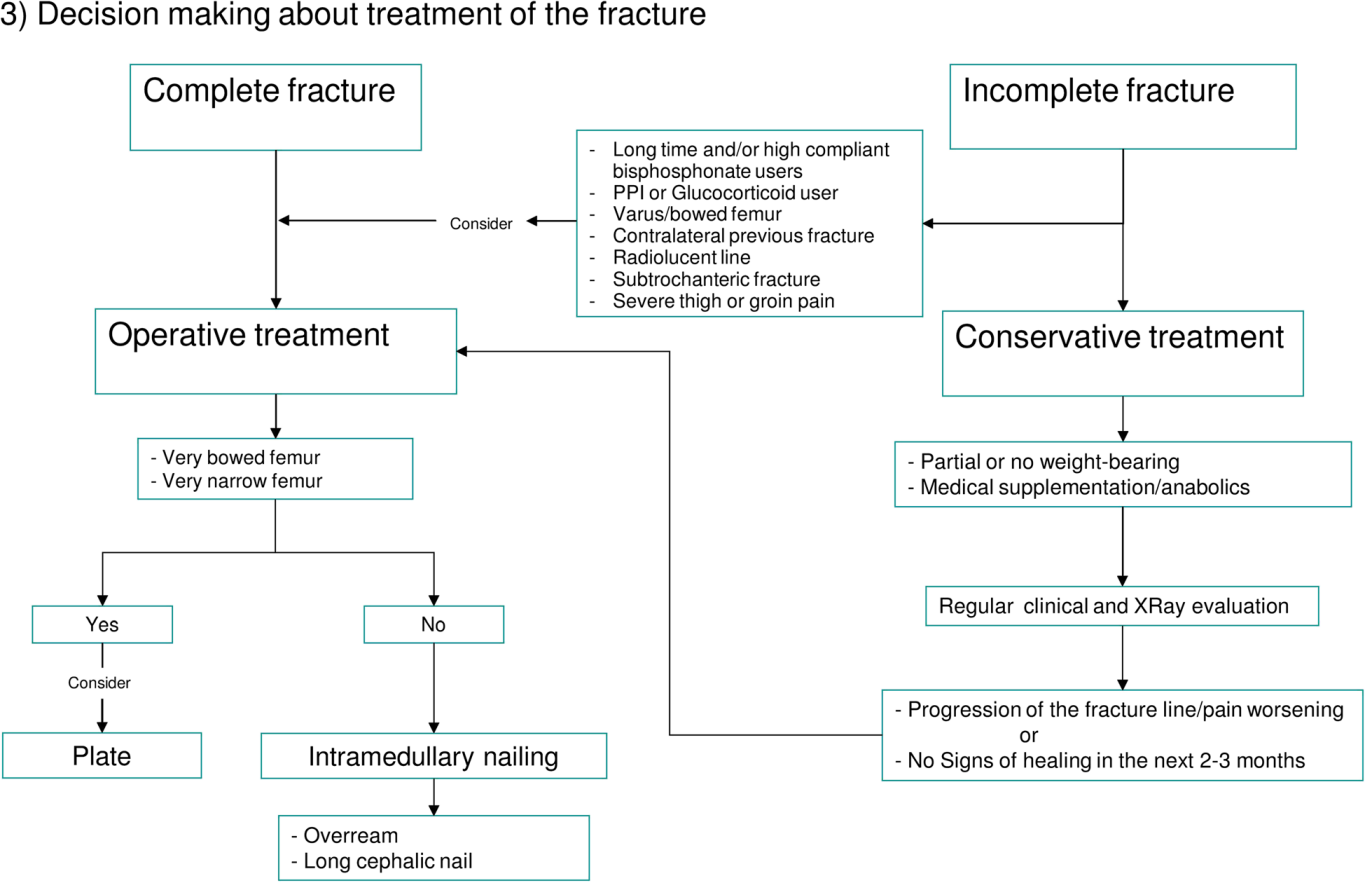
Fracture treatment decision-making

Further assessment of AFF can be done in three steps:

1. Investigation of pathogenic factors of AFF including bone metabolic disorders (Fig. 7)
2. Evaluation of the contralateral femur (Fig. 8)
3. Decision making about treatment of the fracture (Fig. 9)

### Investigation of pathogenic factors of AFF including evaluation of bone turnover (Fig. 7)

We consider that AFF could be differentiated in two major subtypes depending on bone turnover: fractures in individuals with SSBT or without SSBT. In this way, orthopedic surgeons can make a more appropriate diagnosis and perform a better medical and surgical management of these fractures.

A thorough patient history, clinical examination and analysis of appropriate bone biomarkers can offer a general idea of the underlying bone metabolism. The guidelines published by the European Society for Clinical and Economic Aspects of Osteoporosis and Osteoarthritis (ESCEO) and the International Osteoporosis Foundation (IOF) recommend collecting serum calcium and phosphorus levels, intact parathyroid hormone (iPTH), 25-OH- Vitamin D and at least one resorption (i.e. the C-terminal telopeptide, CTX) and one formation bone biomarker (i.e. N-terminal propeptide of type-I procollagen, P1NP or bone alkaline phosphatase) [91]. However, it is advisable to complete the evaluation of bone health through DXA and a complete metabolic assessment, even after hospital discharge.

Being dependent on the results of the aforementioned evaluations, we could distinguish patients in “low turnover”, “normal turnover” and “high turnover”. BRAFF should theoretically occur in low turnover group. However, Giusti et al. found that both bone formation and resorption biomarkers, were in the normal range in most cases (79 and 69.7 % respectively) and were decreased only in a small percentage of cases (14 and 18.2 % respectively) [35]. Anyway, these findings are susceptible to misinterpretation considering that most of these evaluations were obtained around the time of fracture, in a period when the bone was healing and turnover would be expected to be elevated. Thus, the patients with fractures probably had a false-normal turnover, which should be more correctly considered as a hidden low bone turnover.

In case of a low bone turnover AFF, the fracture might be associated to antiresorptive therapy (i.e. BPs) or genetic bone disease (i.e. hypophosphatasia). In this group as well as in case of false-normal turnover, there is a rational to stop antiresorptive therapy, and to consider anabolic drugs according to ASBMR [12]. The relationship between AFF and antiresorptive therapy is most likely related to the mechanism of action of these drugs, both BPs and denosumab [92–98], even if they affect osteoclasts in different ways [99]. BPs bind to hydroxyapatite crystals are phagocyted by osteoclasts promoting their apoptosis, thus inhibiting bone resorption [100]. Denosumab, a fully human monoclonal antibody, binds to RANKL preventing RANK-RANKL interaction, thus inhibiting osteoclast activity [101].

However, medical therapy should be tailored for all patients, particularly in those with “high turnover”, considering that this condition could change the diagnosis of AFF towards other bone disorders (i.e. Paget’s disease of bone).

**Fig. 10:**
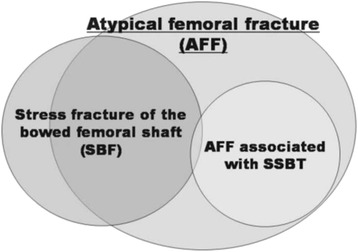
Modified AFF pathogenic scheme proposed by Oh et al. [41, 42] reprinted with permission of authors

Furthermore, some authors have hypothesized that changes in proximal and diaphyseal femoral geometry played a key role as a major risk factor for AFF [36–39]. Oh et al. suggested that tensile stress caused by femoral bowing, contributed towards mechanical failure by modifying the femoral biomechanics, which would account for most cases of AFF (Fig. 10) [41, 42].

### Management of the contralateral femur (Fig. 8)

The treatment decision-making is dependent on the type of fracture (if any) observed in the contralateral femur and the risk of fracture progression. In case of incomplete contralateral fractures, further treatment depends mostly on the associated symptoms. If the patient has thigh or groin pain, prophylactic surgery is advised. On the other hand, when the patient is asymptomatic, conservative treatment may be attempted for the first 2 or 3 months, with strict observation in order to quickly perform prophylactic surgery if signs of fracture progression or non-union occur [14]. In asymptomatic incomplete fractures associated with a simultaneous contralateral complete fracture, prophylactic surgery could be the gold standard to allow early weight bearing, but the ultimate decision depends on patient’s preferences. In individuals with a negative X-ray examination of the contralateral femur, clinical observation should remain strict. If thigh or groin pain appears during follow-up, further investigations such as a bone scan or MRI is highly recommended. If imaging findings are compatible with diagnosis of a fracture, a conservative treatment cycle for up to 2–3 months may be initiated. If pain worsens or becomes persistent, prophylactic surgery should be considered. In this case, fracture healing could be evaluated repeating MRI or bone scans [77]. On the other hand, if these imaging studies show no signs of fracture, a follow, up possibly through serial DXA scans, may be performed. We suggest that in patients who have already sustained an AFF, careful evaluation of DXA scans of the contralateral femur is mandatory, performing a long femur scan, that does not alter proximal femur bone mineral density (BMD) measurements [52, 102].

### Decision making regarding treatment of the fracture (Fig. 9)

Careful evaluation of the femoral geometry can be helpful in order to avoid any of the complications observed with IM nailing. Bridge plating could be useful in patients with very bowed or narrow femora.

Several authors demonstrated the reliability of non-operative treatment that should be adapted to each patient, particularly keeping in mind the bone turnover status [65, 66, 71, 74, 77–81]. The ASBMR task force recommended a trial of conservative treatment in patients with minimal to mild pain, whereas a prophylactic nailing is indicated in case of painful incomplete fracture [25]. Prophylactic nailing seems to be the favorite treatment for incomplete fractures [75, 76, 78, 103, 104], since conservative treatment seems to be less effective [75, 76] and surgery demonstrated to provide faster fracture healing and better pain relief [103]. However, in some patients surgery can be very challenging, and unsuccessful outcomes were reported in some cases [83]. In case of conservative treatment, patients should be followed-up during the next 2 or 3 months, to assess possible fracture progression, worsening symptoms or absence of radiological signs of bone healing, that would make prophylactic surgery necessary [12, 14, 25]. In a review of 14 cases of incomplete fractures managed conservatively, Saleh et al. found that those which presented a radiolucent line (the “dreaded black line”) were the most likely to fail [77]. In another review of 65 incomplete AFF, surgery was indicated more commonly in subtrochanteric fractures than in diaphyseal ones [105].

In our opinion and according to the results of several studies, prophylactic surgery should be the treatment of choice in those patients who are at high risk for progression of the AFF, namely those who are on long-term antiresorptive therapy and/or are highly compliant with this treatment, PPI or glucocorticoid users, individuals with a varus neck-shaft angle or a bowed femur (both in the lateral and anteroposterior plane), patients who have sustained a contralateral AFF, patients with a transverse radiolucent line at standard radiographs, patients with a subtrochanteric fracture, patients with severe or worsening thigh or groin pain, and patients who failed to improve with conservative treatment [14, 35–37, 59, 77, 105]. We provided an operational algorithm for managing AFF that better summarizes the information discussed in the preceding sections (Fig. 6).

Our study has some limitations. The effectiveness of our algorithm could be limited by the lack of evidence of the available literature. However, we chose the scoping review methodology considering that clinical trials represent only the 0.76 % of the literature, limiting thus the utility of systematic reviews and meta-analyses, Instead, in this way we analyzed and summarized the vast majority of the literature. On the other hand, no standardized tools to define the quality of included studies were used.

Further studies with a higher level of evidence are needed in order to address several issues that remain unresolved. Indeed, we identified three aspects which we believe require further investigation: the metabolic characterization of the fracture (which in our opinion could be an important guide toward diagnosis and treatment), the identification of further AFF risk assessment tools, and the role of drug anabolic therapy and other non-pharmacological interventions to enhance AFF healing (i.e. therapeutic ultrasound or others form of mechanical energy).

## Conclusions

Atypical femoral fractures are a rare entity of femoral stress fracture that occur in the region between subtrochanteric and supracondylar areas, and that initially involve the lateral femoral cortex. Large efforts have been made in order to characterize these fractures, from their clinical presentation and imaging pattern to their pathogenesis. However, the level of evidence in the available literature is mostly poor, particularly regarding the choice of treatment.

## Abbreviations

AFF: atypical femoral fractures
ASBMR: American society for bone and mineral research
BMD: bone mineral density
BPs: bisphosphonates
BRAFF: BPs-related AFF
CT: computed tomography
CTX: C-terminal telopeptide
DALYs: disability and life-years lost
DXA: dual-energy x-ray absorptiometry
EMA: European medicines agency
ESCEO: European society for clinical and economic aspects of osteoporosis and osteoarthritis
FAERS: FDA adverse event reporting system
FDA: food and drug administration
IM: intramedullary
IOF: international osteoporosis foundation
iPTH: intact parathyroid hormone
MRI: magnetic resonance imaging
P1NP: N-terminal propeptide of type-I procollagen
PPI: proton pump inhibitors
SSBT: severe suppressed bone turnover
